# Curcumin for the clinical treatment of rheumatoid arthritis: a systematic review and meta-analysis of placebo-controlled randomized clinical trials

**DOI:** 10.3389/fimmu.2025.1726157

**Published:** 2026-01-12

**Authors:** Yihua Fan, Zhiqiang Yi, Shijie Mao, Jialu Wen, Jiwei Zhang, Qiang Zhang, Ruihan Liu

**Affiliations:** 1 Department of Rheumatism and Immunity, Hospital of Chengdu University of Traditional Chinese Medicine, Chengdu, Sichuan, China; 2 School of Clinical Medicine, Chengdu University of Traditional Chinese Medicine, Chengdu, Sichuan, China; 3 Bazhong Hospital of Traditional Chinese Medicine, Bazhong, Sichuan, China; 4 Department of Scientific Research, Shapingba Hospital affiliated to Chongqing University, Shapingba District People’s Hospital of Chongqing, Chongqing, China

**Keywords:** curcumin, meta-analysis, randomized controlled trials, rheumatoid arthritis, systematic review

## Abstract

**Background:**

The efficacy and safety of curcumin in the treatment of rheumatoid arthritis (RA) remain controversial. We therefore conducted a meta-analysis to evaluate the efficacy and safety of curcumin in RA.

**Methods:**

We searched PubMed, Embase, the Cochrane Library and Web of Science for relevant literature published up to July 30, 2025. The Cochrane risk of bias tool was used to assess bias in the included trials, and the Grade of Recommendations Assessment, Development and Evaluation (GRADE) framework was applied to evaluate the certainty of evidence. Meta-analyses were performed using Review Manager 5.3.

**Results:**

Six studies involving 244 participants were included. The meta-analysis showed that curcumin significantly improved the following outcomes: American College of Rheumatology (ACR) 20 response (SMD = 4.35, 95%CI(2.22, 6.47), *P* < 0.0001; evidence certainty: very low), disease activity score(DAS-28) (SMD = -3.40, 95%CI(-5.29, -1.50), *P* = 0.0004; very low), erythrocyte sedimentation rate (ESR) level (SMD = -3.72, 95%CI(-5.26, -2.18), *P* < 0.00001; very low), C-reactive protein (CRP) level (SMD = -2.91, 95%CI(-4.42, -1.39), *P* = 0.0002; very low), visual analogue scale (VAS) score (SMD = -5.65, 95%CI(-6.95, -4.34), *P* < 0.00001; very low), tender joint count (TJC) (SMD = -2.84, 95%CI(-4.47, -1.22), *P* = 0.0006; very low), swollen joint count (SJC) (SMD = -4.11, 95%CI(-6.19, -2.03), *P* = 0.0001; very low), and rheumatoid factor (RF) level (SMD = -3.82, 95%CI(-4.62, -3.02), *P* < 0.00001; low).

**Conclusion:**

Current evidence suggests that curcumin has a significant therapeutic effect on RA. However, given the limitations of this meta-analysis, future multicenter, large-sample, placebo-controlled randomized trials are warranted to further verify its efficacy and safety.

**Systematic Review Registration:**

https://www.crd.york.ac.uk/PROSPERO/view/CRD420251147977, identifier CRD420251147977.

## Introduction

Rheumatoid arthritis (RA) is an autoimmune disease characterized by chronic inflammatory joint lesions (1, 2). Its underlying pathology involves multiple factors and typically presents with joint pain, swelling, stiffness and impaired function, significantly impairing patients’ quality of life (3). In recent years, the global prevalence of RA has risen steadily. In 2021, an estimated 17.9 million individuals were affected worldwide, with the disease increasingly occurring in younger populations (4). From 1990 to 2021, incidence among individuals aged 20-54 years increased by 81.21%, while RA-related mortality rose by 2.97% (5). In China, the number of RA patients continues to grow annually, showing distinct age and sex distribution patterns. The highest risk occurs in the 55-59- year age group, and women are affected approximately twice as often as men (6). Modeling projections suggest that the incidence and prevalence of RA in China are expected to keep rising through 2042 (7).

The pathogenesis of RA remains incompletely elucidated but is believed to involve genetic predisposition, environmental triggers, infectious agents, and dysregulated immune activation (8, 9). The disease process is marked by synovial inflammation, destruction of articular cartilage and bone, and eventual joint deformity and functional loss (10, 11). Current treatment aims to alleviate symptoms, improve joint function, delay disease progression, and enhance patients’ quality of life (12). Commonly used agents include non-steroidal anti-inflammatory drugs, glucocorticoids and disease-modifying antirheumatic drugs such as methotrexate, leflunomide, sulfasalazine, etc. (12, 13). However, these therapies are often associated with adverse effects-including gastrointestinal disturbances, and some patients exhibit poor treatment responses (14). Furthermore, long-term use of glucocorticoids can also lead to complications such as osteoporosis and diabetes (15). Consequently, identifying safer and more effective treatment strategies remains an important focus of medical research.

Curcumin, a natural polyphenol derived from the rhizome of *Curcuma longa* (turmeric) (16), exhibits a range of biological activities including anti-inflammatory, antioxidant and anti-tumor properties (17, 18). In recent years, increasing attention has been directed toward its potential application in RA management (19). By inhibiting the production and release of inflammatory mediators and modulating the immune response (20), curcumin may beneficially influence the inflammatory processes underlying RA (21).

Several studies have reported that curcumin can reduce inflammatory markers and improve clinical symptoms in RA patients (22–24). A previous meta-analysis indicated that curcumin supplementation may improve inflammatory levels and clinical manifestations in RA (25), and additional randomized controlled trials have been published in recent years. However, existing findings on curcumin for RA remain inconsistent (26). While some studies demonstrate significant efficacy (27–29), others report limited or non-significant effects (30, 31). Given these divergent results, an updated systematic synthesis of the evidence is warranted to accurately and comprehensively evaluate the safety and efficacy of curcumin in RA treatment. Therefore, this study aimed to perform a meta-analysis to thoroughly assess the efficacy and safety of curcumin in patients with RA, thereby providing evidence to inform clinical practice.

## Methods

This meta-analysis was conducted in accordance with the Preferred Reporting Items for Systematic Reviews and Meta-Analyses (PRISMA) 2020 guidelines and has been registered on the PROSPERO platform (Registration Number: CRD420251147977).

### Search strategy

We systematically searched PubMed, Embase, the Cochrane Library, and Web of Science for randomized controlled trials (RCTs) investigating curcumin treatment in patients with RA, from database inception through July 30, 2025. The search was restricted to English-language publications. Key search terms included: “curcumin,” “rheumatoid arthritis,” “RA,” “randomized controlled trial,” and “randomized.” The full search strategy for each database is provided in **Supplementary Material** (**Supplementary Tables S1**-**S4**).

### Inclusion criteria

Studies were selected based on the PICOS framework:

P(Population): Patients diagnosed with RA, irrespective of age, sex, or ethnicity;

I(Intervention): Curcumin in any formulation or dose;

C(Comparison): Placebo;

O(Outcome): The primary outcome was the American College of Rheumatology 20 (ACR 20) response; Secondary outcomes included the Disease activity score-28(DAS-28), erythrocyte sedimentation rate (ESR), visual analogue scale (VAS), C-reactive protein (CRP) level, tender joint count (TJC), swollen joint count (SJC), rheumatoid factor (RF) level, and incidence of adverse events.

S(Study design): RCTs.

### Exclusion criteria

We excluded: (1) animal studies; (2) case reports, conference abstracts, and reviews; (3) duplicate publications; (4) articles for which the full text was unavailable; and (5) studies with incomplete outcome data.

### Data extraction

Two independent reviewers screened the retrieved records and subsequently assessed the full-text articles against the eligibility criteria. Any discrepancies were resolved through discussion or, if necessary, by consultation with a third reviewer. Data from the included studies were extracted independently by the same two reviewers and cross-checked for accuracy.

### Risk of bias assessment

The methodological quality of the included RCTs was evaluated using the Cochrane Risk of Bias tool, which assesses bias across seven domains: random sequence generation, allocation concealment, blinding of participants and personnel, blinding of outcome assessment, incomplete outcome data, selective reporting, and other potential biases.

### Certainty of the evidence

The overall certainty of evidence for each meta-analyzed outcome was judged using the Grading of Recommendations Assessment, Development and Evaluation (GRADE) framework. Evidence was rated by considering risk of bias, inconsistency, indirectness, imprecision, and publication bias, and was classified into one of four categories: high, moderate, low, or very low.

### Statistical analysis

All analyses were performed using Review Manager software (version 5.3). For continuous outcomes measured on different scales or using different assays, the standardized mean difference (SMD) with 95% confidence interval (CI) was calculated as the summary measure. The magnitude of the SMD was interpreted as follows: 0.20 = small, 0.50 = moderate, and 0.80 = large. Heterogeneity among studies was assessed using the χ^2^ test and the *I^2^* statistic. A random-effects model was employed if significant heterogeneity was present (*P* ≤ 0.10 for χ^2^ or *I^2^* > 50%); otherwise, a fixed-effect model was used. Subgroup analyses were planned based on curcumin dosage form, dose, and geographic region of the study. Sensitivity analyses were conducted by sequentially removing each study to test the robustness of the pooled results. Publication bias was evaluated visually using funnel plots.

## Results

### Search results

**Figure 1:**
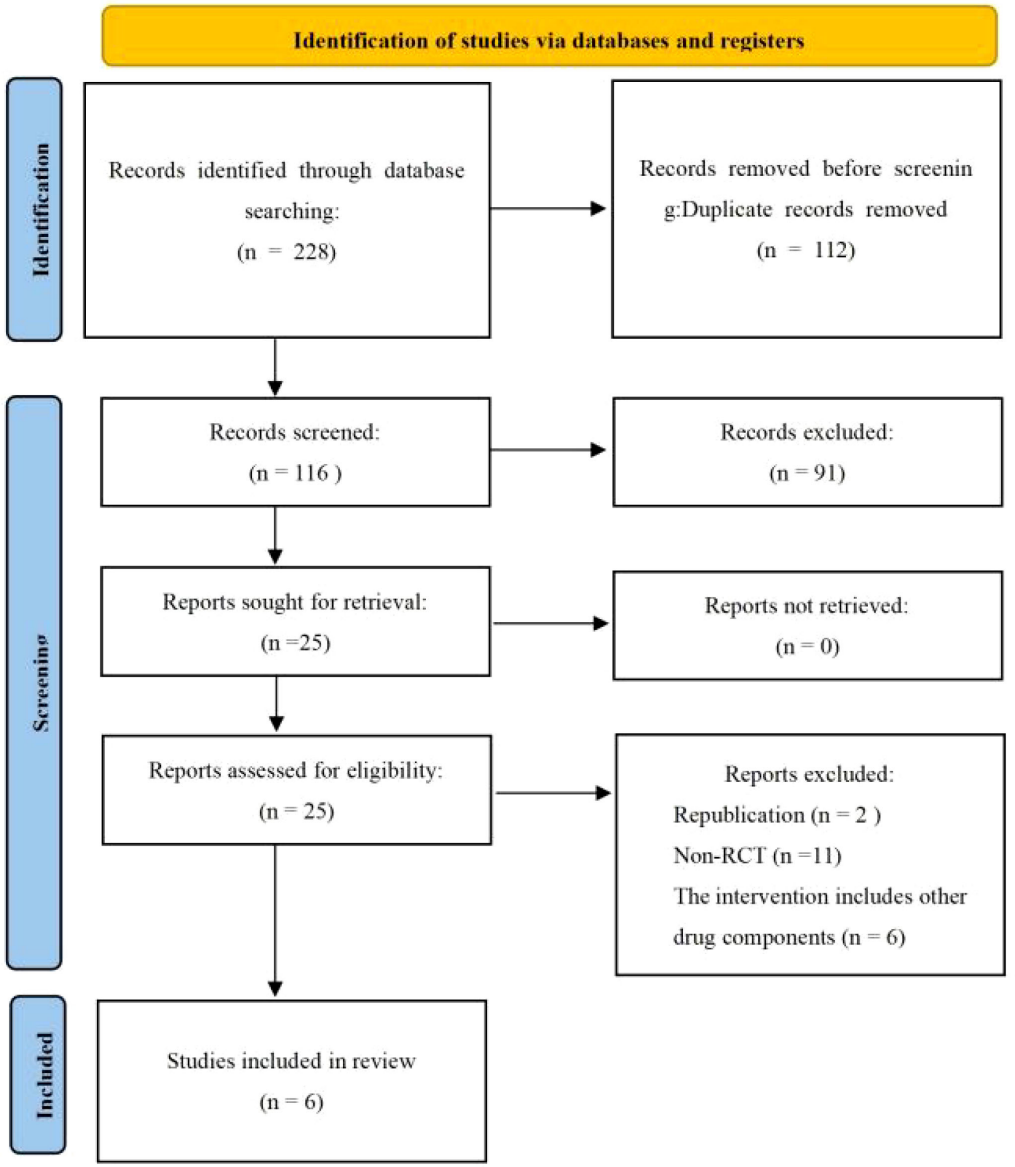
Guidelines flow diagram.

A total of 228 records were initially identified. After removing 112 duplicates using EndNote software and conducting multiple rounds of screening, six studies (27–29, 31–33) were finally included in the meta-analysis (**Figure 1**; **Supplementary Material Table S5**).

### Study characteristics

The six included studies (27–29, 31–33) involved a total of 244 participants: 133 in the curcumin groups and 111 in the placebo groups. Individual trial sample sizes ranged from 24 to 61 participants. Two studies were three-arm trials; for the purpose of analysis, these were split into two separate two-arm comparisons. Four trials were conducted in India and the remaining two in Iran. The dosage of curcumin varied from 40 mg to 500 mg daily, and the intervention duration ranged from 8 to 12 weeks. The baseline characteristics of the included studies are summarized in **Table 1**.

**Table 1:** The basic characteristics included in the trial.

References	Country	Registration number	Design	Research time	Sample size	Age(Years)	Interventions	Dose	Duration (week)
Amalraj et al., 2017 (27)	India	–	RCT	–	12	36.7 ± 10.7	Curcumin	250 mg	12
					12	38.3 ± 5.8	Curcumin	500 mg	
					12	39.6 ± 8.8	Placebo	–	
Jacob et al., 2019 (28)	India	–	RCT	–	8	18-65	Hydrogenated curcuminoids formulation	250 mg	12
					8	18-65	Hydrogenated curcuminoids formulation	500 mg	
					8	18-65	Placebo	–	
Javadi et al., 2019 (31)	Iran	RCT 2017011614925N	RCT	November 2016 to May 2017	24	53.71 ± 2.75	Curcumin nanomicelle	40 mg	12
					25	56.28 ± 2.5	Placebo		
Khamar et al., 2024 (32)	Iran	IRCT20 220 306 054 203N1	RCT	August 2022 to April 2023	15	43.73 ± 2.60	Curcumin nanomicelle	40 mg	12
					15	45.47 ± 2.64	Placebo	–	
Pourhabibi-Zarandi et al., 2024 (29)	Iran	IRCT20100408003664N24	RCT	June 2020 to November 2020	22	50.68 ± 9.93	Curcumin	500 mg	8
					22	50.36 ± 9.70	Placebo	–	
Rezaieyazdi et al., 2023 (33)	Iran	IRCT20170812035630N1	RCT	2017 to 2018	32	22-71	Curcumin	80 mg	12
					29	22-71	Placebo	–	

### Risk of bias assessment

**Figure 2:**
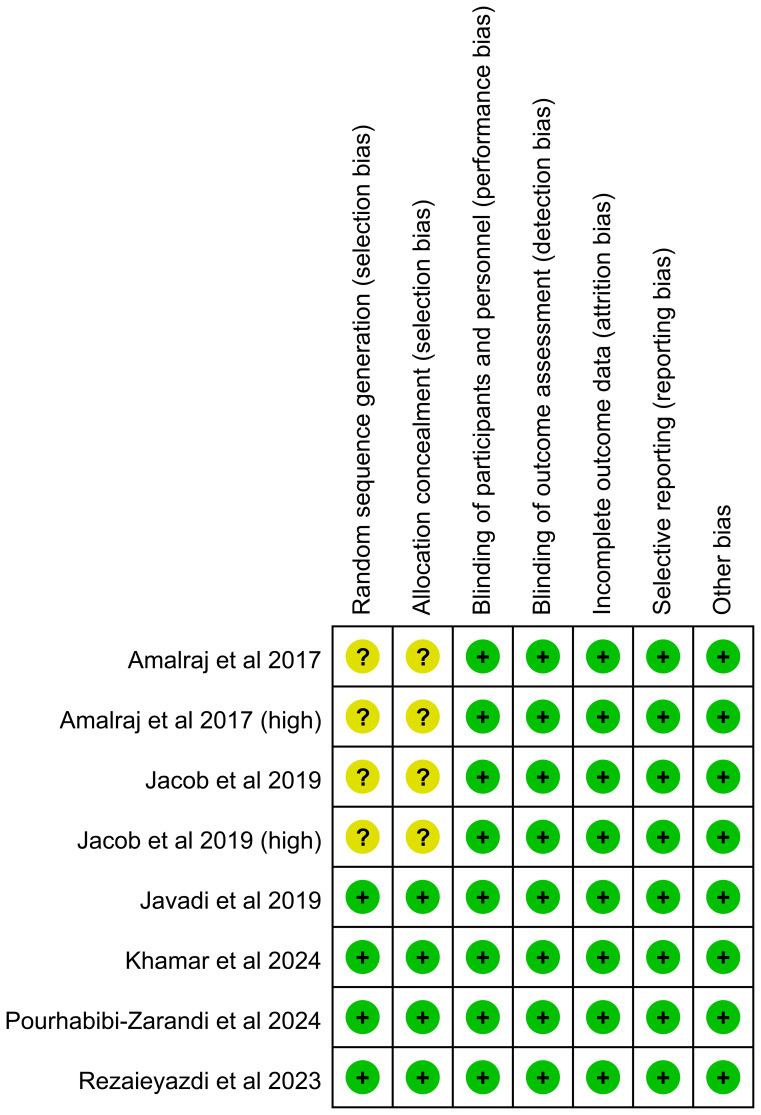
Risk of bias summary.

The risk of bias assessment indicated that the randomization methods and allocation concealment were unclear in two studies. All other domains were judged to be at low risk of bias (**Figure 2**; **Supplementary Material Table S6**).

### Meta-analysis

#### American college of rheumatology 20 score

**Figure 3:**
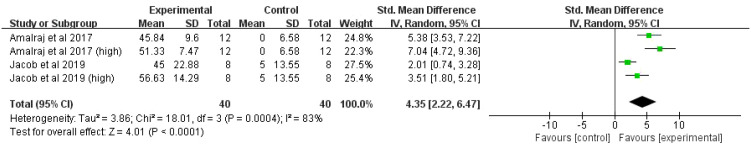
Forest plot of the effect of curcumin treatment on ACR 20 response in RA patients.

Two studies comprising four comparisons and 60 participants reported this outcome. Considerable heterogeneity was observed (*I^2^* = 83%, *P* = 0.0004), and a random-effects model was applied. The meta-analysis showed that curcumin significantly improved the ACR 20 response rate compared with placebo (SMD = 4.35, 95% CI(2.22, 6.47), *P* < 0.0001) (**Figure 3**).

##### Disease activity score-28

**Figure 4:**
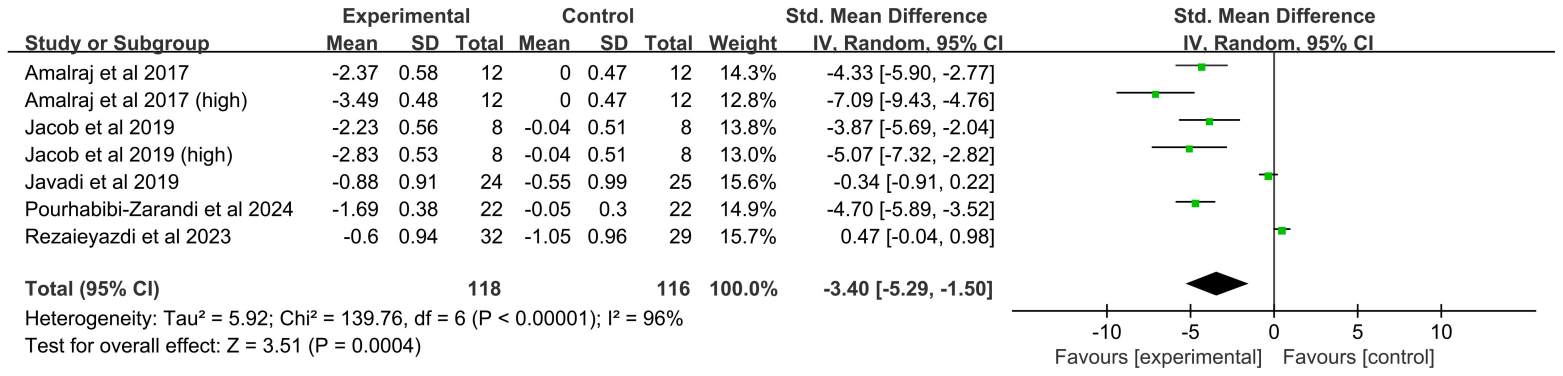
Forest plot of the effect of curcumin treatment on DAS-28 score in RA patients.

Five studies (seven comparisons, 214 participants) were included. High heterogeneity was present (*I^2^* = 96%, *P* < 0.00001), leading to the use of a random-effects model. The analysis demonstrated that curcumin significantly reduced the DAS-28 compared with placebo (SMD = -3.40, 95% CI (-5.29, -1.50), *P* = 0.0004) (**Figure 4**).

##### Visual analogue scale

**Figure 5:**
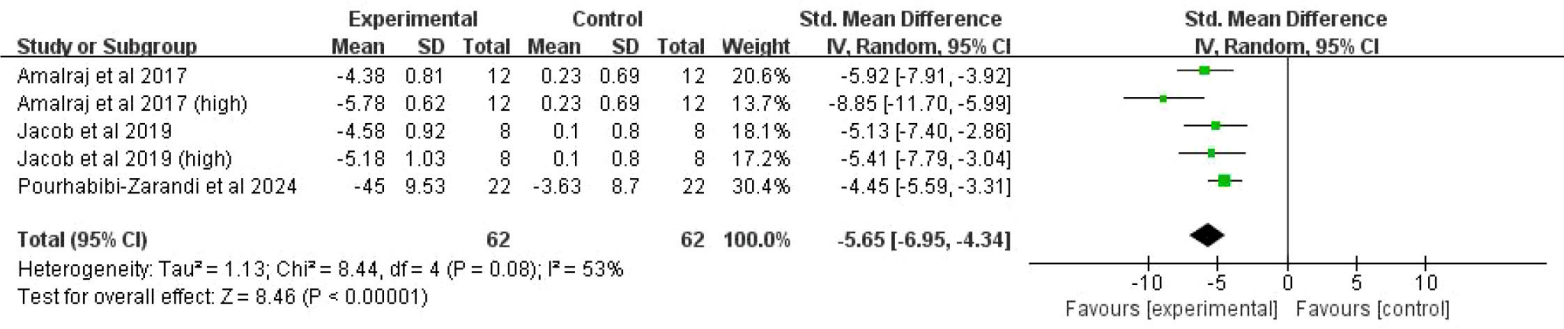
Forest plot of the effect of curcumin treatment on VAS score in RA patients.

Data from three studies (five comparisons, 104 participants) were pooled. Moderate heterogeneity was detected (*I^2^* = 53%, *P* = 0.08), and a random-effects model was employed. The results indicated that curcumin significantly lowered the VAS pain score versus placebo (SMD = -5.65, 95% CI(-6.95, -4.34), *P* < 0.00001) (**Figure 5**).

##### Erythrocyte sedimentation rate

**Figure 6:**
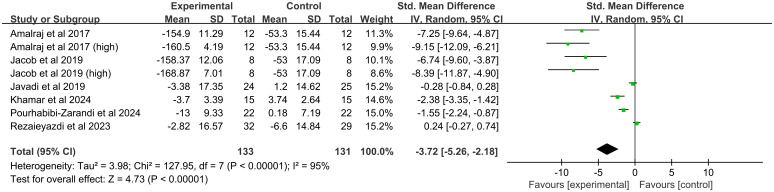
Forest plot of the effect of curcumin treatment on ESR level in RA patients.

All six studies (eight comparisons, 244 participants) reported ESR levels. Substantial heterogeneity was found (*I^2^* = 95%, *P* < 0.00001), and a random-effects model was used. The meta-analysis revealed a significant reduction in ESR with curcumin treatment (SMD = -3.72, 95% CI(-5.26, -2.18), *P* < 0.00001) (**Figure 6**).

##### C-reactive protein

**Figure 7:**
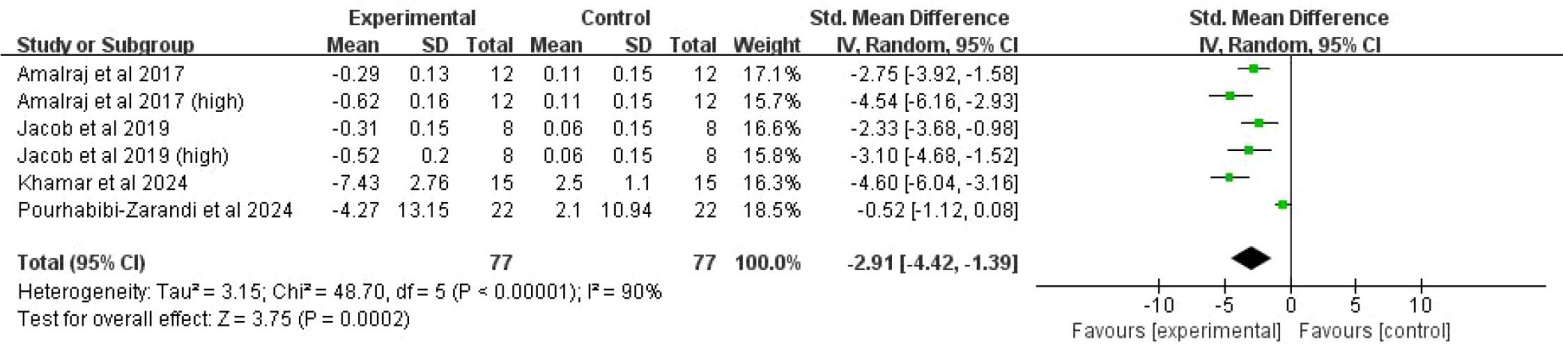
Forest plot of the effect of curcumin treatment on CRP level in RA patients.

Four studies (six comparisons, 134 participants) contributed data. High heterogeneity was observed (*I^2^* = 90%, *P* < 0.00001), warranting a random-effects model. The pooled results showed that curcumin significantly decreased CRP levels compared to placebo (SMD = -2.91, 95% CI(-4.42, -1.39), *P* = 0.0002) (**Figure 7**).

##### Tender joint count

**Figure 8:**
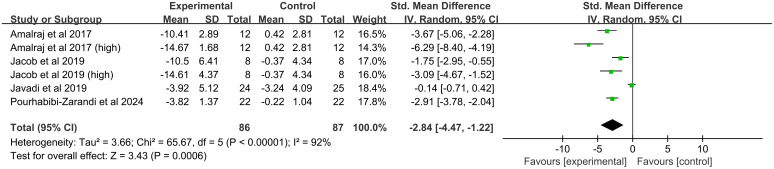
Forest plot of the effect of curcumin treatment on TJC in RA patients.

Four studies (six comparisons, 153 participants) were analyzed. Significant heterogeneity existed (*I^2^* = 92%, *P* < 0.00001), and a random-effects model was applied. The analysis indicated that curcumin significantly reduced the TJC (SMD = -2.84, 95% CI(-4.47, -1.22), *P* = 0.0006) (**Figure 8**).

##### Swollen joint count

**Figure 9:**
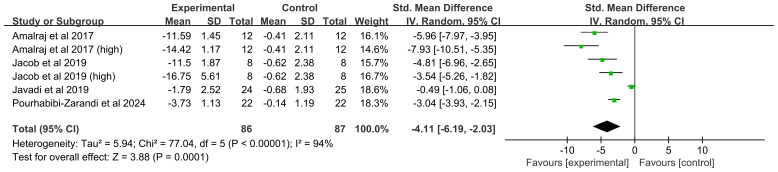
Forest plot of the effect of curcumin treatment on SJC in RA patients.

Four studies (six comparisons, 153 participants) were analyzed. Heterogeneity was high (*I^2^* = 94%, *P* < 0.00001), leading to the use of a random-effects model. The meta-analysis found that curcumin significantly decreased the SJC (SMD = -4.11, 95% CI(-6.19, -2.03), *P* = 0.0001) (**Figure 9**).

##### Rheumatoid factor

**Figure 10:**
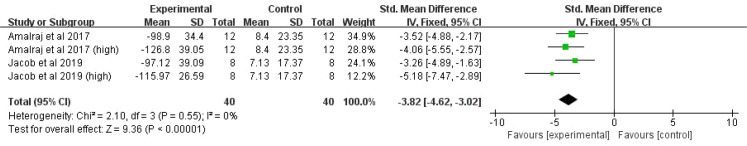
Forest plot of the effect of curcumin treatment on RF level in RA patients.

Two studies (four comparisons, 60 participants) reported RF levels. No significant heterogeneity was detected (*I^2^* = 0%, *P* = 0.55), so a fixed-effects model was used. The results showed that curcumin significantly lowered RF levels compared with placebo (SMD = -3.82, 95% CI(-4.62, -3.02), *P* < 0.00001) (**Figure 10**).

##### Adverse events

No adverse events occurred in any of the included trials.

##### Subgroup analysis

Subgroup analyses were performed based on curcumin dosage form (Hydrogenated curcuminoids formulation vs. Curcumin vs. Curcumin nanomicelle), dosage (≤250 mg vs. >250 mg) and study region (India vs. Iran). Detailed results are presented in **Table 2**.

**Table 2:** Subgroup analysis summary results.

Subgroup analysis	The number of studies included	Outcomes	Heterogeneity		Model	Meta-analysis	
			*P*	*I^2^*		SMD (95%CI)	*P*
Dosage form							
Hydrogenated curcuminoids formulation	2	DAS-28	0.41	0%	Fixed	**-4.35(-5.76, -2.93)**	**<0.00001**
Curcumin	4		<0.00001	97%	Random	**-3.83(-7.49, -0.17)**	**0.04**
Country							
India	4		0.16	41%	Fixed	**-4.80(-5.76, -3.85)**	**<0.00001**
Iran	3		<0.00001	97%	Random	-1.44(-3.61, 0.72)	0.19
Dose							
≤ 250 mg	4		<0.00001	94%	Random	**-1.80(-3.51, -0.08)**	**0.04**
> 250 mg	3		0.2	38%	Fixed	**-5.17(-6.13, -4.21)**	**<0.00001**
Dosage form							
Hydrogenated curcuminoids formulation	2	ESR	0.47	0%	Fixed	**-7.40(-9.61, -5.19)**	**<0.00001**
Curcumin	4		<0.00001	96%	Random	**-3.95(-6.56, -1.34)**	**0.003**
Curcumin nanomicelle	2		0.0002	93%	Random	-1.29(-3.35, 0.76)	0.22
Country							
India	4		0.65	0%	Fixed	**-7.76(-9.18, -6.34)**	**<0.00001**
Iran	4		<0.00001	91%	Random	-0.94(-2.00, 0.12)	0.08
Dose							
≤ 250 mg	5		<0.00001	94%	Random	**-2.72(-4.47, -0.97)**	**0.002**
> 250 mg	3		<0.00001	95%	Random	**-6.21(-12.02, -0.40)**	**0.04**
Dosage form							
Hydrogenated curcuminoids formulation	2	CRP	0.47	0%	Fixed	**-2.66(-3.68, -1.63)**	**<0.00001**
Curcumin	3		<0.00001	93%	Random	**-2.51(-4.85, -0.18)**	**0.03**
Country							
India	4		0.2	35%	Fixed	**-3.04(-3.74, -2.34)**	**<0.00001**
Iran	2		<0.00001	96%	Random	**-2.91(-4.42, -1.39)**	**0.0002**
Dose							
≤ 250 mg	3		0.06	65%	Random	**-3.19(-4.48, -1.90)**	**<0.00001**
> 250 mg	3		<0.00001	93%	Random	**-2.64(-5.28 -0.00)**	**0.05**
Dosage form							
Hydrogenated curcuminoids formulation	2	VAS	0.87	0%	Fixed	**-5.27(-6.91, -3.63)**	**<0.00001**
Curcumin	3		0.01	76%	Random	**-6.10(-8.37, -3.82)**	**<0.00001**
Dose							
≤ 250 mg	2		0.61	0%	Fixed	**-5.57(-7.07, -4.07)**	**<0.00001**
> 250 mg	3		0.02	75%	Random	**-5.96(-8.35, -3.58)**	**<0.00001**
Dosage form							
Hydrogenated curcuminoids formulation	2	Tender joint count	0.18	43%	Fixed	**-2.24(-3.20, -1.29)**	**<0.00001**
Curcumin	3		0.01	77%	Random	**-4.05(-5.71, -2.38)**	**<0.00001**
Country							
India	4		0.003	79%	Random	**-3.55(-5.22, -1.89)**	**<0.0001**
Iran	2		<0.00001	96%	Random	-1.51(-4.21, 1.20)	0.28
Dose							
≤ 250 mg	3		<0.00001	92%	Random	-1.78(-3.84, 0.28)	0.09
> 250 mg	3		0.01	96%	Random	**-3.89(-5.65, -2.13)**	**<0.0001**
Dosage form							
Hydrogenated curcuminoids formulation	2	Swollen joint count	0.37	0%	Fixed	**-4.03(-5.38, -2.69)**	**<0.00001**
Curcumin	3		0.0002	88%	Random	**-5.46(-8.45, -2.48)**	**0.0003**
Country							
India	4		0.04	65%	Random	**-5.41(-7.17, -3.64)**	**<0.0001**
Iran	2		<0.00001	96%	Random	-1.74(-4.24, 0.76)	0.17
Dose							
≤ 250 mg	3		<0.00001	95%	Random	-3.66(-7.59, 0.27)	0.07
> 250 mg	3		0.002	84%	Random	**-4.55(-6.91, -2.19)**	**0.0002**
Dosage form							
Hydrogenated curcuminoids formulation	2	Rheumatoid Factor	0.18	45%	Fixed	**-3.91(-5.23, -2.58)**	**<0.00001**
Curcumin	2		0.6	0%	Fixed	**-3.77(-4.77, -2.76)**	**<0.00001**
Dose							
≤ 250 mg	2		0.81	0%	Fixed	**-3.42(-4.46, -2.38)**	**<0.00001**
> 250 mg	2		0.42	0%	Fixed	**-4.39(-5.64, -3.14)**	**<0.00001**
Dosage form							
Hydrogenated curcuminoids formulation	2	American College of Rheumatology 20	0.17	47%	Fixed	**2.54(1.52, 3.56)**	**<0.00001**
Curcumin	2		0.27	17%	Fixed	**6.02(4.58, 7.47)**	**<0.00001**
Dose							
≤ 250 mg	2		0.003	88%	Random	**3.62(0.33, 6.92)**	**0.03**
> 250 mg	2		0.02	83%	Random	**5.18(1.72, 8.64)**	**0.003**

DAS-28, disease activity score-28; VAS, visual analogue scale; ESR, erythrocyte sedimentation rate;CRP, C-reactive protein.The bold values are those with P ≤ 0.05, indicating statistical differences.

#### American college of rheumatology 20 response

Both hydrogenated curcuminoids (SMD = 2.54, 95%CI(1.52, 3.56, *P* < 0.00001); and standard curcumin formulations (SMD = 6.02, 95%CI(4.58, 7.47), *P* < 0.00001) significantly improved the ACR20 response. Benefits were also observed for both dosage categories (≤ 250 mg: SMD = 3.62, 95%CI (0.33, 6.92), *P* = 0.03; > 250 mg: SMD = 5.18, 95%CI (1.72, 8.64), *P* = 0.003).

##### Disease activity score-28

A significant reduction in DAS-28 was seen with hydrogenated curcuminoids (SMD = -4.35, 95%CI(-5.76, -2.93, *P* < 0.00001)and curcumin (SMD = -3.83, 95%CI(-7.49, -0.17), *P* = 0.04). When analyzed by region, a significant effect was found in studies from India (SMD = -4.80, 95%CI(-5.76, -3.85), *P* < 0.00001)) but not in those from Iran (SMD = -1.44, 95%CI(-3.61, 0.72), *P* = 0.19).). Both lower and higher doses were effective (≤ 250 mg: SMD = -1.80,95%CI(-3.51, -0.08), *P* = 0.04; > 250 mg: SMD = -5.17, 95%CI(-6.13, -4.21), *P* < 0.00001).

##### Visual analogue scale

Both formulation types significantly reduced VAS scores (hydrogenated curcuminoids: SMD = -5.27, 95%CI(-6.91, -3.63, *P* < 0.00001); curcumin: SMD = -6.10, 95%CI(-8.37, -3.82), *P* < 0.00001). A significant benefit was also observed for both dose ranges (≤ 250 mg: SMD = -5.57,95%CI(-7.07, -4.07), *P* < 0.00001; > 250 mg: SMD = -5.96, 95%CI(-8.35, -3.58), *P* < 0.00001).

##### Erythrocyte sedimentation rate

ESR levels were significantly reduced by hydrogenated curcuminoids (SMD = -7.40, 95%CI(-9.61, -5.19, *P* < 0.00001) and curcumin (SMD = -3.95, 95%CI(-6.56, -1.34), *P* = 0.003). A significant effect was found in Indian studies (SMD = -7.76, 95%CI(-9.18, -6.34), *P* < 0.00001) but not in Iranian studies (SMD = -0.94, 95%CI(-2.00, 0.12), *P* = 0.08). Both dosage subgroups showed efficacy (≤ 250 mg: SMD = -2.72,95%CI(-4.47, -0.97), *P* = 0.002; > 250 mg: SMD = -6.21, 95%CI(-12.02, -0.40), *P* = 0.04).

##### C-reactive protein

CRP levels were significantly lowered by both hydrogenated curcuminoids (SMD = -2.66, 95%CI(-3.68, -1.63, *P* < 0.00001) and curcumin (SMD = -2.51, 95%CI(-4.85, -0.18), *P* = 0.03). Significant reductions were observed in studies from both India (SMD = -3.04, 95%CI(-3.74, -2.34), *P* < 0.00001) and Iran (SMD = -2.91, 95%CI(-4.42, -1.39, *P* = 0.0002). A significant effect was found for lower doses (≤ 250 mg: SMD = -3.19,95%CI(-4.48, -1.90), *P* < 0.00001)), whereas the effect for higher doses (>250 mg) was of borderline statistical significance (SMD = -2.64, 95%CI(-5.28 -0.00), *P* = 0.05).

##### Tender joint count

TJC was significantly improved by both formulation types (hydrogenated curcuminoids: SMD = -2.24, 95%CI(-3.20, -1.29, *P* < 0.00001); curcumin: SMD = -4.05, 95%CI(-5.71, -2.38), *P* < 0.00001). A significant reduction was seen in Indian studies (SMD = -3.55, 95%CI(-5.22, -1.89), *P* < 0.0001) but not in Iranian studies (SMD = -1.51, 95%CI(-4.21, 1.20), *P* = 0.28). While higher doses (>250 mg) showed a significant benefit (SMD = -3.89, 95%CI(-5.65, -2.13), *P* < 0.0001), the effect for lower doses (≤ 250 mg) was not statistically significant (SMD = -1.78,95%CI(-3.84, 0.28), *P* = 0.09).

##### Swollen joint count

SJC was significantly reduced by both hydrogenated curcuminoids (SMD = -4.03, 95%CI(-5.38, -2.69, *P* < 0.00001) and curcumin (SMD = -5.46, 95%CI(-8.45, -2.48), *P* = 0.0003). A significant effect was observed in Indian studies (SMD = -5.41, 95%CI(-7.17, -3.64), *P* < 0.0001) but not in Iranian studies (SMD = -1.74, 95%CI(-4.24, 0.76), *P* = 0.17). Higher doses (>250 mg) showed a significant benefit (SMD = -4.55, 95%CI (-6.91, -2.19), *P* = 0.0002), whereas the effect for lower doses (≤250 mg) was not statistically significant (SMD = -3.66,95%CI (-7.59, 0.27), *P* = 0.07).

##### Rheumatoid factor

RF levels were significantly reduced by both hydrogenated curcuminoids (SMD = -3.91, 95%CI(-5.23, -2.58, *P* < 0.00001) and curcumin (SMD = -3.77, 95%CI(-4.77, -2.76), *P* < 0.00001). Significant effects were observed for both dosage subgroups (≤ 250 mg: SMD = -3.42, 95%CI (-4.46, -2.38), *P* < 0.00001; > 250 mg: SMD = -4.39, 95%CI (-5.64, -3.14), *P* < 0.00001).

##### Sensitivity analysis

**Figure 11:**
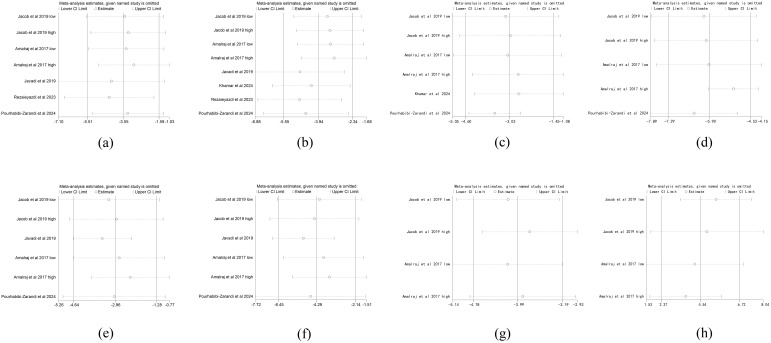
Sensitivity analysis. **(a)** Disease activity score-28, **(b)**Erythrocyte sedimentation rate, **(c)** C-reactive protein, **(d)** Visual analogue scale, **(e)** Tender joint count, **(f)** Swollen joint count, **(g)** Rheumatoid factor,and **(h)** American college of rheumatology 20 response.

A leave-one-out sensitivity analysis was performed for each outcome. The results indicated that the pooled effect estimates for all outcomes remained robust and were not substantially altered by the removal of any single study (**Figure 11**).

##### Publication bias

**Figure 12:**
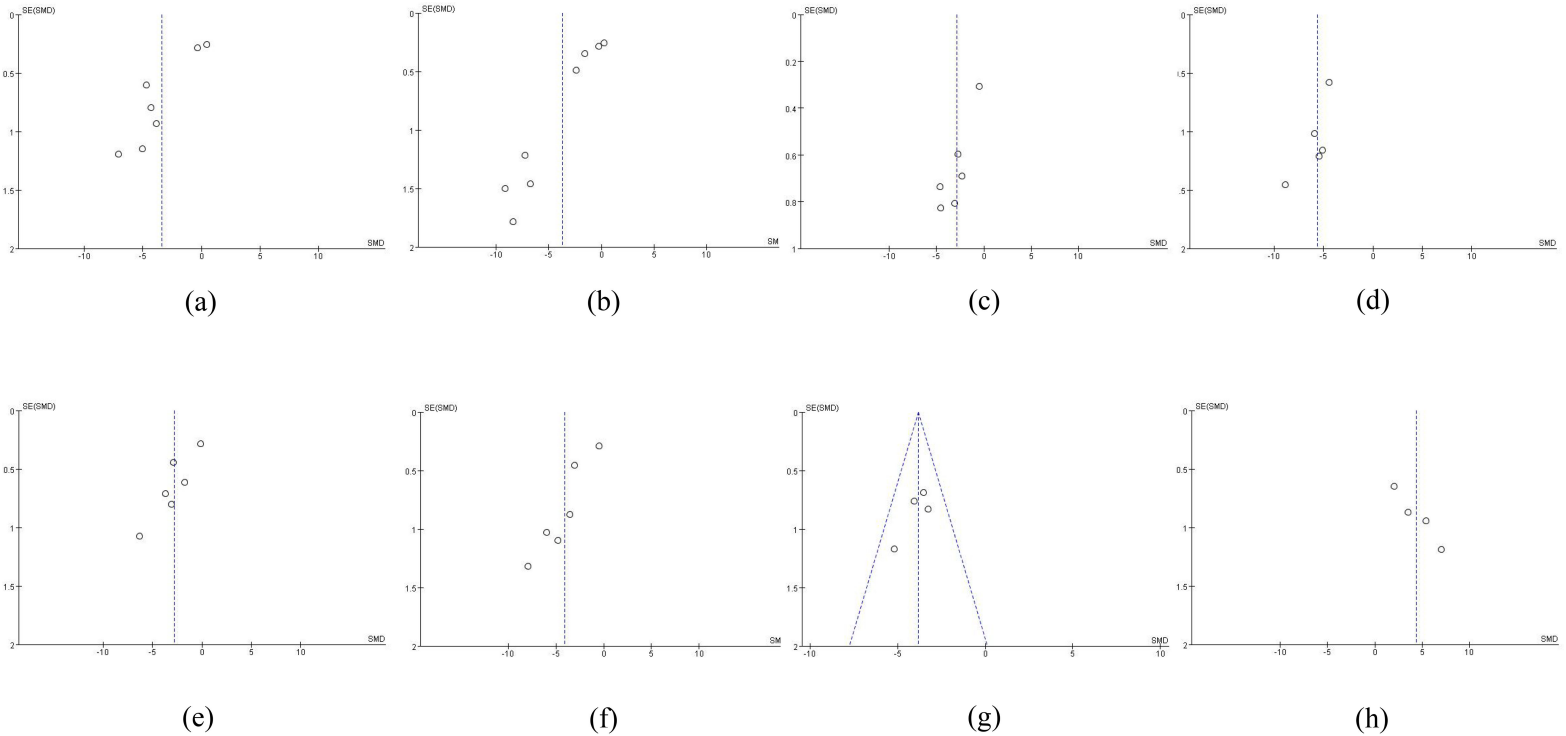
Publication bias analysis. **(a)** Disease activity score-28, **(b)**Erythrocyte sedimentation rate, **(c)** C-reactive protein, **(d)** Visual analogue scale, **(e)** Tender joint count, **(f)** Swollen joint count, **(g)** Rheumatoid factor,and **(h)** American college of rheumatology 20 response.

Visual inspection of the funnel plot suggested asymmetry, indicating the potential presence of publication bias (**Figure 12**). However, as the number of included studies was fewer than ten, the statistical power of this test is limited, and the results should therefore be interpreted with caution.

##### Certainty of evidence

The certainty of evidence for all outcomes was systematically evaluated using the GRADE framework. The two studies contributing to several outcomes did not describe allocation concealment or blinding procedures and were therefore rated as having a serious risk of bias. Substantial heterogeneity (*I²* > 50%) was present for the outcomes DAS-28, ESR, CRP, VAS, TJC, SJC, and ACR 20, leading to a rating of serious inconsistency for these measures. All outcomes were based on direct evidence, so indirectness was not a concern. Imprecision was rated as serious for all outcomes due to the small total sample sizes and wide confidence intervals. Publication bias was also judged to be serious based on the asymmetrical funnel plot. Consequently, the certainty of evidence for one outcome was rated as low, while for all others it was rated as very low (**Table 3**).

**Table 3:** GRADE systematic evaluation of evidence quality.

Outcomes	Quality assessment					Intervention/Control	SMD(95%CI)	Certainty of the evidence
	Risk of bias	Inconsistency	Indirectness	Imprecision	Publication bias			
DAS-28	Limitations^1^	Serious^2^	Not serious	Serious^3^	Serious^4^	118/116	-3.40(-5.29, -1.50)	Very low:⊕◯◯◯
ESR	Limitations^1^	Serious^2^	Not serious	Serious^3^	Serious^4^	133/131	-3.72(-5.26, -2.18)	Very low:⊕◯◯◯
CRP	Limitations^1^	Serious^2^	Not serious	Serious^3^	Serious^4^	77/77	-2.91(-4.42, -1.39)	Very low:⊕◯◯◯
VAS	Limitations^1^	Serious^2^	Not serious	Serious^3^	Serious^4^	62/62	-5.65(-6.95, -4.34)	Very low:⊕◯◯◯
Tender joint count	Limitations^1^	Serious^2^	Not serious	Serious^3^	Serious^4^	86/87	-2.84(-4.47, -1.22)	Very low:⊕◯◯◯
Swollen joint count	Limitations^1^	Serious^2^	Not serious	Serious^3^	Serious^4^	86/87	-4.11(-6.19, -2.03)	Very low:⊕◯◯◯
Rheumatoid Factor	Limitations^1^	Not serious	Not serious	Serious^3^	Serious^4^	40/40	-3.82(-4.62, -3.02)	Low:⊕⊕◯◯
American College of Rheumatology 20	Limitations^1^	Serious^2^	Not serious	Serious^3^	Serious^4^	40/40	4.35(2.22, 6.47)	Very low:⊕◯◯◯

^1^Some RCTs did not mention using the randomized scheme hiding or randomized grouping; ^2^Significant heterogeneity;^3^The study sample size is small; ^4^The funnel plot was asymmetrical.

## Discussion

This meta-analysis comprehensively evaluated the efficacy of curcumin in patients with RA. The results demonstrated that curcumin significantly improved multiple outcome measures, including DAS-28, ESR, CRP, VAS score, tender and swollen joint counts, RF levels, and the ACR20 response rate. These findings provide preliminary support for curcumin as a potential adjunctive supplement in RA management. However, these apparently positive results must be interpreted with caution and balanced against the notable limitations of the present analysis.

Our findings are consistent with several previous studies reporting that curcumin improves inflammatory markers and clinical symptoms in RA patients (34). However, other trials have failed to observe significant differences (31, 33). For instance, one study noted that while curcumin reduced VAS scores and swollen joint counts in the short term, its effects DAS-28 and TJC were not significant (33). Another trial reported an improvement in joint pain that did not reach statistical significance (31). Variations in the formulation and dosage of curcumin across studies may partly explain these discrepancies. Some studies employed relatively low doses, which may be insufficient to elicit a marked therapeutic effect (27, 28). Furthermore, the inherently low oral bioavailability of curcumin is a key factor limiting its efficacy (35). Several included trials (31, 32), did not utilize bioavailability-enhancing technologies, which likely restricted systemic absorption and activity.

Notably, our subgroup analyses suggest that hydrogenated curcuminoid formulations, which typically offer improved bioavailability and stability, may demonstrate greater efficacy. Furthermore, higher curcumin doses (>250 mg/day) were associated with superior outcomes across several indicators, particularly for improving joint pain and swelling. This aligns with evidence that the anti-inflammatory action of curcumin is dose-dependent within a certain range (36). These observations underscore that optimizing the formulation and dosage of curcumin is critical for maximizing its clinical benefit. Future research should focus on elucidating the specific mechanisms and determining the optimal dosing regimens for different curcumin preparations in RA. While no adverse events were reported in the trials included in this analysis, a systematic assessment of safety remains largely absent. There was a lack of active monitoring and standardized reporting for potential side effects, such as gastrointestinal discomfort or effects on liver function. Establishing a clear safety profile for curcumin-particularly at high doses or in specific formulations-is crucial for patients with RA who require long-term management.

Curcumin is believed to exert its effects through multiple anti-inflammatory, antioxidant, and immunomodulatory pathways. It can inhibit the IκB kinase/NF-κB and PI3K/Akt signaling pathways, thereby reducing the production of pro-inflammatory cytokines (37). It also downregulates the expression of cyclooxygenase-2 (COX-2) and inducible nitric oxide synthase (iNOS), decreasing prostaglandin and nitric oxide synthesis (16). Additionally, curcumin suppresses the activation of immune cells such as macrophages and reduces inflammatory cell infiltration (38, 39). Beyond its anti-inflammatory properties, curcumin exhibits potent antioxidant activity by scavenging free radicals and mitigating cellular oxidative stress (40). It further enhances cellular antioxidant defense by upregulating the expression of endogenous antioxidant enzymes (41). With regard to pain relief, curcumin may modulate pain signaling by inhibiting transient receptor potential vanilloid 1 (TRPV1) receptor activity and enhancing the function of endogenous opioid pathways (42, 43). Specifically, it has been shown to inhibit the transient receptor potential vanilloid type 1 (TRPV1) receptor, thereby reducing nociceptive signaling (42). Additionally, curcumin can modulate opioid receptor activity and potentiate the analgesic effects of endogenous opioid peptides (43). These multifaceted mechanisms collectively provide a theoretical foundation for the therapeutic application of curcumin in RA.

Several important limitations of this analysis must be acknowledged. First, the enrolled patient populations were predominantly from Asian countries (India and Iran), which may limit the generalizability of findings to European or other ethnic groups. Second, variations in the manufacturing processes of the curcumin preparations used across studies could influence their bioactivity and clinical effects. Third, the overall certainty of evidence, as assessed by the GRADE framework, was rated as very low. This stems primarily from the small number of included trials, the limited total sample size, and the substantial heterogeneity observed between studies. Although subgroup analyses were conducted to explore sources of heterogeneity, its persistent presence undermines the reliability of the pooled effect estimates and suggests that the observed treatment differences may be strongly influenced by various confounding factors beyond the intervention itself. Consequently, the current results should be viewed as preliminary and suggestive rather than conclusive. Fourth, while none of the included trials reported adverse events, systematic safety evaluation was virtually absent. There was a lack of active monitoring and standardized reporting for potential side effects such as gastrointestinal discomfort or hepatotoxicity. Establishing a comprehensive safety profile for curcumin-particularly at higher doses or in specific formulations-is crucial for its potential long-term use in RA management. Fifth, the effect of curcumin as an adjunctive therapy may interact with background medications or depend on baseline disease activity, but insufficient data precluded such analyses. Finally, the limited number of studies restricted more extensive subgroup analyses, and funnel plot asymmetry suggested potential publication bias, which should be considered when interpreting the results. Given the current “very low” certainty of evidence, it is premature to recommend the routine clinical use of curcumin for RA. Clinicians should be aware of its potential benefits while recognizing the substantial uncertainty in the evidence base. Conclusions derived from this analysis require cautious and circumspect application.

## Conclusion

Existing evidence suggests that curcumin may have a significant therapeutic effect on RA. While these findings are informative, the serious limitations of the included studies preclude definitive recommendations. Further large-sample, multi-center, and long- long-term RCTs are warranted to verify the efficacy and safety of curcumin in RA and to more precisely define its potential role in clinical practice.

## Data Availability

The original contributions presented in the study are included in the article/**Supplementary Material**. Further inquiries can be directed to the corresponding authors.
