# Supplementary material for: Curcumin for the clinical treatment of rheumatoid arthritis: a systematic review and meta-analysis of placebo-controlled randomized clinical trials
Source: Front Immunol. 2026 Jan 12;16:1726157. doi: 10.3389/fimmu.2025.1726157 (PMC12832973; doi:10.3389/fimmu.2025.1726157)
Supplement: Supplementary file 1 [file Table1.docx]

**Supplementary Material**

| **Table S1. Search strategies in Pubmed** | |
| --- | --- |
| Number | Search terms |
| #1 | "Rheumatoid Arthritis"[MeSH] OR "Rheumatoid Arthritis"[Title/Abstract] OR"Arthritis, Rheumatoid"[Title/Abstract] OR "RA"[Title/Abstract] |
| #2 | "Curcumin"[MeSH] OR "Curcumin"[Title/Abstract] OR"Turmeric"[Title/Abstract] OR "Curcuma longa"[Title/Abstract] OR "Diferuloylmethane"[Title/Abstract] |
| #3 | "Randomized Controlled Trials as Topic"[MeSH] OR "Randomized Controlled Trial"[Publication Type] OR"Random*"[Title/Abstract] OR "Clinical Trials, Randomized"[Title/Abstract] OR "Trials, Randomized Clinical"[Title/Abstract] OR "Controlled Clinical Trials, Randomized"[Title/Abstract] OR "Randomized Controlled Trial"[Title/Abstract] OR "Randomized Controlled Trial"[Title/Abstract] OR "Randomized Controlled Trial"[Title/Abstract] OR "Randomized Controlled Trials"[Title/Abstract] OR "Clinical Trials as Topic"[MeSH] OR "Clinical Trial as Topic"[Title/Abstract] OR "Clinical Trial"[Title/Abstract] OR "Clinical Trials"[Title/Abstract] OR "Controlled Clinical Trials as Topic"[MeSH] OR "Controlled Clinical Trials"[Title/Abstract] OR "Controlled Clinical Trial"[Title/Abstract] OR "Clinical Trials, Controlled as Topic"[Title/Abstract] |
| #4 | (#1) AND (#2) AND (#3) |

| **Table S2. Search strategies in Web of Science** | |
| --- | --- |
| Number | Search terms |
| #1 | TS = ("Rheumatoid Arthritis" OR RA OR "Rheumatoid Arthritides") |
| #2 | TS = ("Curcumin" OR "Curcumins" OR "Turmeric Extract") |
| #3 | TS = ("Randomized Controlled Trial" OR "Randomized trial" OR "RCT" OR "Controlled trial") |
| #4 | (#1) AND (#2) AND (#3) |

| **Table S3. Search strategies in Embase** | |
| --- | --- |
| Number | Search terms |
| #1 | ('rheumatoid arthritis' OR 'rheumatoid arthritis' OR 'RA' OR 'rheumatoid arthritides') |
| #2 | ('curcumin' OR 'curcumin' OR 'curcumins' OR 'turmeric extract') |
| #3 | ('randomized controlled trial' OR 'RCT') |
| #4 | (#1) AND (#2) AND (#3) |

| **Table S4. Search strategies in Cochrane Library** | |
| --- | --- |
| Number | Search terms |
| #1 | (''Rheumatoid Arthritis'' OR ''RA'' OR ''Rheumatoid Arthritides'') |
| #2 | (''Curcumin'' OR ''Curcumins'' OR ''Turmeric Extract'') |
| #3 | (''Randomized Controlled Trial'' OR ''RCT'') |
| #4 | (#1) AND (#2) AND (#3) |

| **Table S5. Reports excluded record** | | |
| --- | --- | --- |
|  | Reports | Exclusion reasons |
| 1 | Efficacy and safety of curcumin in maintaining remission during disease-modifying antirheumatic drug withdrawal in rheumatoid arthritis at 52 weeks: a phase III double-blind, randomized placebo-controlled trial | The intervention included other components. |
| 2 | The Effect of Curcumin Supplementation on Clinical Factors in Patients with Rheumatoid Arthritis | Non-RCT. |
| 3 | Therapeutic Potential of Curcumin with and without Strengthening Exercises in Improving Rheumatoid Arthritis | The intervention included other components. |
| 4 | EFFICACY AND SAFETY OF CURCUMIN IN MAINTAINING REMISSION DURING DISEASE MODIFYING ANTI RHEUMATIC DRUG WITHDRAWAL IN RHEUMATOID ARTHRITIS AT 52 WEEKS: PHASE III DOUBLE-BLIND RANDOMIZED PLACEBO CONTROLLED TRIAL | Republication. |
| 5 | Efficacy of Nanocurcumin with application of Iontophoresis on Inflammatory arthritis patients. | The intervention included other components. |
| 6 | Efficacy of a mouthwash containing essential oils and curcumin as an adjunct to nonsurgical periodontal therapy among rheumatoid arthritis patients with chronic periodontitis: A randomized controlled trial | The intervention included other components. |
| 7 | Phytoconstituents as pharmacotherapeutics in rheumatoid arthritis: challenges and scope of nano/submicromedicine in its effective delivery | Non-RCT. |
| 8 | ANTIOXIDANTS AND ANTIINFLAMMATORY DIETARY SUPPLEMENTS FOR OSTEOARTHRITIS AND RHEUMATOID ARTHRITIS | Non-RCT. |
| 9 | Curcumin in Autoimmune and Rheumatic Diseases | Non-RCT. |
| 10 | Curcumin nanoparticles and the therapeutic potential of curcumin for musculoskeletal disorders | Non-RCT. |
| 11 | Clinical studies with curcumin | Non-RCT. |
| 12 | Oral Administration of Nano-Emulsion Curcumin in Mice Suppresses Inflammatory-Induced NFκB Signaling and Macrophage Migration | Non-RCT. |
| 13 | Clinical Utility of Curcumin Extract | Non-RCT. |
| 14 | Raging the War Against Inflammation With Natural Products | Non-RCT. |
| 15 | The Clinical Use of Curcumin for the Treatment of Rheumatoid Arthritis: A Systematic Review of Clinical Trials | Non-RCT. |
| 16 | Phytotherapy as an adjunct to the treatment of rheumatoid arthritis - a systematic review of clinical trials | Non-RCT. |
| 17 | STUDY THE EFFECTS OF ANTI-INFLAMMATORY CURCUMEX CAPSULES CONTAINING THREE PLANTS (GINGER, CURCUMIN AND BLACK PEPPER) IN PATIENTS WITH ACTIVE RHEUMATOID ARTHRITIS | The intervention included other components. |
| 18 | A commercialized dietary supplement alleviates joint pain in community adults: a double-blind, placebo-controlled community trial | The intervention included other components. |
| 19 | Effects of curcumin supplementation on metabolic parameters,inflammatory factors and obesity values in women with rheumatoid arthritis: A randomized, double-blind, placebo controlled clinical trial | Republication |

| **Table S6. Risk of bias** | | | | | | | |
| --- | --- | --- | --- | --- | --- | --- | --- |
| Study, year | Random sequence generation | Allocation concealment | Blinding of participants and personnel | Blinding of outcome assessment | Incomplete outcome data | Selective Reporting | Other bias |
| Amalraj et al 2017 | Unclear | Unclear | Low risk | Low risk | Low risk | Low risk | Low risk |
| Jacob et al 2019 | Unclear | Unclear | Low risk | Low risk | Low risk | Low risk | Low risk |
| Javadi et al 2019 | Low risk | Low risk | Low risk | Low risk | Low risk | Low risk | Low risk |
| Khamar et al 2024 | Low risk | Low risk | Low risk | Low risk | Low risk | Low risk | Low risk |
| Pourhabibi-Zarandi et al 2024 | Low risk | Low risk | Low risk | Low risk | Low risk | Low risk | Low risk |
| Rezaieyazdi et al 2023 | Low risk | Low risk | Low risk | Low risk | Low risk | Low risk | Low risk |
